# Comparative Analysis of Expression Profiles of Panicle Development among Tolerant and Sensitive Rice in Response to Drought Stress

**DOI:** 10.3389/fpls.2017.00437

**Published:** 2017-03-29

**Authors:** Haibin Wei, Chen Chen, Xiaosong Ma, Yu Zhang, Jing Han, Hanwei Mei, Shunwu Yu

**Affiliations:** Shanghai Agrobiological Gene CenterShanghai, China

**Keywords:** drought tolerance, gene-based association mapping study, functional analysis, panicle, *Oryza sativa*

## Abstract

Water deficit caused a serious threat to crops, especially panicle development at reproductive growth phase. We investigated grain yield components and gene expression profiles of panicle among tolerant and sensitive rice in response to drought stress. Panicle morphologies exhibited that secondary branches per panicle were more severely affected as compared to primary branches per panicle. Moreover, grain weight per panicle showed significant decrease for both tolerant and sensitive varieties except for MILT1444. Expression profile analysis revealed that 783 differentially expressed genes (DEGs) were identified to be drought-induced from young panicles in 2 cm length. Hierarchical clustering indicated that 76.8% of DEGs were up-regulated for all six rice varieties, and the percentage of down-regulated genes was higher in sensitive group than tolerant group. Biological process category revealed that the shared Gene Ontology (GO) terms were involved in response to abiotic stimulus and stress, whereas the specific GO terms in tolerant group were identified as regulation of biological quality, homeostatic process, cell growth, anatomical structure morphogenesis and development, and the unique terms in sensitive varieties were identified as lipid metabolic process and secondary metabolic process. Furthermore, the gene-based association analysis narrowed down list of DEGs, and four genes common to all six varieties were selected as candidate for breeders. Together, we found several shared and distinct biological processes between tolerant and sensitive varieties, and candidate stress-responsive genes. These findings provided insight into functional mechanisms regulating drought stress response in panicle development and may also help to crop tolerant improvement.

## Introduction

Drought is one of the main environmental challenges for cereal crop growth and productivity, and has impact on global food security (Boyer, 1982; Luo et al., 2009). This is especially urgent for negative effects of future climate change on agricultural productivity, while food production needs to double in the coming decades (Ray et al., 2013). Drought disaster that occurs during reproductive phase often causes more severe yield loss than that during vegetative phase (Takai et al., 2006; Xing and Zhang, 2010). The development of drought tolerant rice cultivars is one of the major challenges currently for sustainable agriculture (Luo, 2010). The identification of stress-responsive genes can greatly facilitate the improvement of rice breeding with enhanced drought tolerance.

The adaptation aspects of drought stress are complex and involve numerous plant processes, including physiological, metabolic and molecular adaptations (Chaves et al., 2003; Fischer et al., 2012). In rice, yield components, such as panicle length, primary/secondary branches per panicle, seed setting rate, and grain weight per panicle, are significantly reduced by drought (Muthurajan et al., 2011). For the reproductive phase, panicle and spikelet developments as key factors of grain yield formation, and small changes of developing panicle can severely affect yield components in water deficit (Sikuku et al., 2012). The previous studies have revealed that genes of photosynthesis and developmental regulation in the leaf are remarkably inhibited by drought in the reproductive stage when plants are in the water-deficit situation (Luo et al., 2009; Jin et al., 2013; Johnson et al., 2014). Additionally, rice natural populations have been used to identify genes related to drought response by genome-wide association study (GWAS) (Yu et al., 2012; Lou et al., 2015). The identified genetic mutations (SNPs) may help further elucidate the mechanisms through which DEGs enhanced the potential drought tolerance.

High-throughput techniques, such as microarray and deep sequencing, have been widely used to monitor expression profiling of plants under stress condition in the last dozen years (Rabbani et al., 2003; Zhang et al., 2005; Matsui et al., 2008; Lenka et al., 2011). Recent advances in molecular biology of rice have been reported in temporal and spatial protein and transcript expression profiling to understand various physiological and molecular processes (Wang et al., 2011). Gene microarray revealed that Ghd7 regulated yield traits through modulating panicle architecture independent of the heading date and was involved in abiotic and biotic stress-response pathways (Weng et al., 2014). Moreover, stress-responsive genes were tissue- or stage-specific regulated (Wang et al., 2011). Hence, this study was formulated to understand the effect of drought on development of young panicle and molecular changes leading to grain yield losses.

To achieve a more comprehensive understanding of coordinating mechanism between panicle development and drought tolerance, we combined gene expression profiling and gene-based association analysis within tolerant and sensitive rice. In the present study, we collected six rice varieties with different level of drought tolerance and investigated changes in gene expression in panicle by rice gene microarrays under drought stress and well watered conditions. The differentially expressed genes (DEGs) offered insight into important processes on protection of panicle development against drought environment in rice. Furthermore, the gene-based association mapping study discovered that almost half of DEGs were harbored association SNPs signals. The drought tolerant rice varieties and association analysis are propitious to narrow the candidate genes, which have more potential value of rice breeding and enable high efficiency of genetic approaches to improve crop tolerance.

## Results

### Drought Stress Phenotypic Traits of Panicle Development among Contrasting Rice Varieties

**FIGURE 1:**
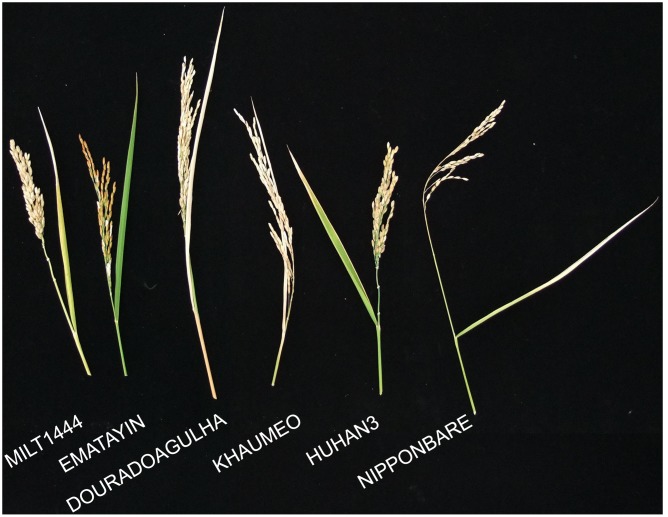
**Rice morphological characters at mature stages after 2 weeks of drought treatment at later tillering stage**. MILT444, EMATAYIN, DOURADOAGULHA, and HUHAN3, was drought resistant with high drought index, while KHAUMEO and NIPPONBARE were drought sensitive with small drought index.

The drought index as the drought-tolerant capacity has been investigated in our previous studies (Yu et al., 2012). Six rice varieties were selected according to the well-characterized drought index (**Table 1**), including drought tolerant varieties, MILT1444, EMATAYIN, DOURADOAGULHA and HUHAN3, and drought sensitive varieties, KHAUMEO and NIPPONBARE. After successive drought treatment at later tillering stage, phenotypic evaluations of panicles at maturity stage were conducted. The phenotypic traits of panicle under drought stress observed that contrasting rice varieties differed for a range of characters, including panicle shape and size; grain shape, size, and color (**Figure 1**). On the whole, the drought tolerant varieties showed greener and larger spikelets than drought sensitive varieties due to a longer delayed heading and flowering time by the inhibitory effect of drought stress in tolerant varieties than that in sensitive varieties.

**Table 1:** The drought index, origins and types of tested cultivated rice.

Name	Drought index	Origin	*Indica*/*japonica*
MILT1444	1.45	Philippines	*japonica*
EMATAYIN	1.63	Myanmar	*japonica*
DOURADOAGULHA	1.88	Indian	*Indica*
KHAUMEO	0.51	Vietnam	*Indica*
HUHAN3	2.13	China	*japonica*
NIPPONBARE	0.54	Japan	*japonica*

**FIGURE 2:**
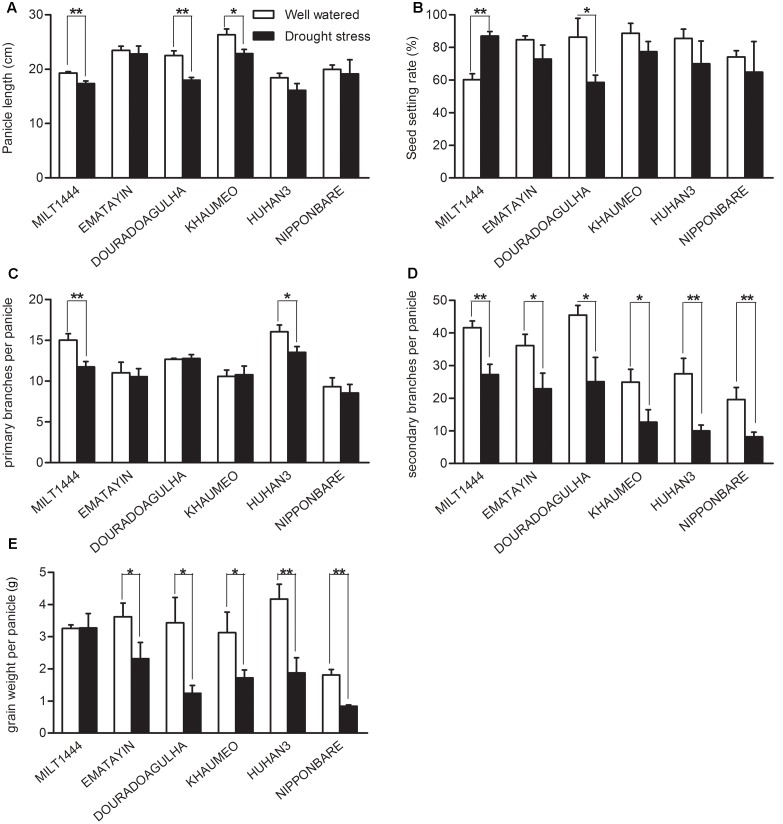
**Comparison of some important agronomic traits between well watered and drought stress conditions with the ripening time. (A)** The decreased panicle length were detected more significant difference in MILT1444, DOURADOAGULHA, and KHAUMEO. **(B)** The seeding setting rate almost decreased under drought stress except MILT1444 with increased trend. **(C,D)** Primary branches per panicle were less frequently declined by water stress than secondary branches per panicle in six varieties. **(E)** The reduction of grain weight per panicle was significant in all varieties except MILT1444. Each value in **(A–E)** represents the mean ± SD of three independent biological replicates. Significant levels are marked with asterisks, ^∗^*P* < 0.05 and ^∗∗^*P* < 0.01 versus well watered.

Yield components were also examined for detailed comparison between well watered and drought stress conditions (**Figure 2**). The panicle lengths were obviously decreased in MILT1444 and DOURADOAGULHA in drought tolerant varieties, while length decrease was observed in KHAUMEO in drought sensitive varieties (**Figure 2A**). The changes of seed setting rate and primary branches per panicle in six varieties were comparatively less under drought stress (**Figures 2B,C**). Importantly, secondary branches per panicle were more frequently declined by drought stress (**Figure 2D**). The phenotypic changes in rice led to a reduction of grain weight per panicle and finally decreased the yield (**Figure 2E**). The MILT1444 with high drought index showed no significant decline of grain weight per panicle, which could be due to over 80% of the seeding setting rate still being kept under drought stress (**Figures 2B,E**). The results demonstrated that drought tolerance varieties, such as MILT1444, can reduce grain yield losses when compared to drought sensitive varieties in response to drought stress.

### Differential Gene Expression in Panicles under Drought Treatment

**FIGURE 3:**
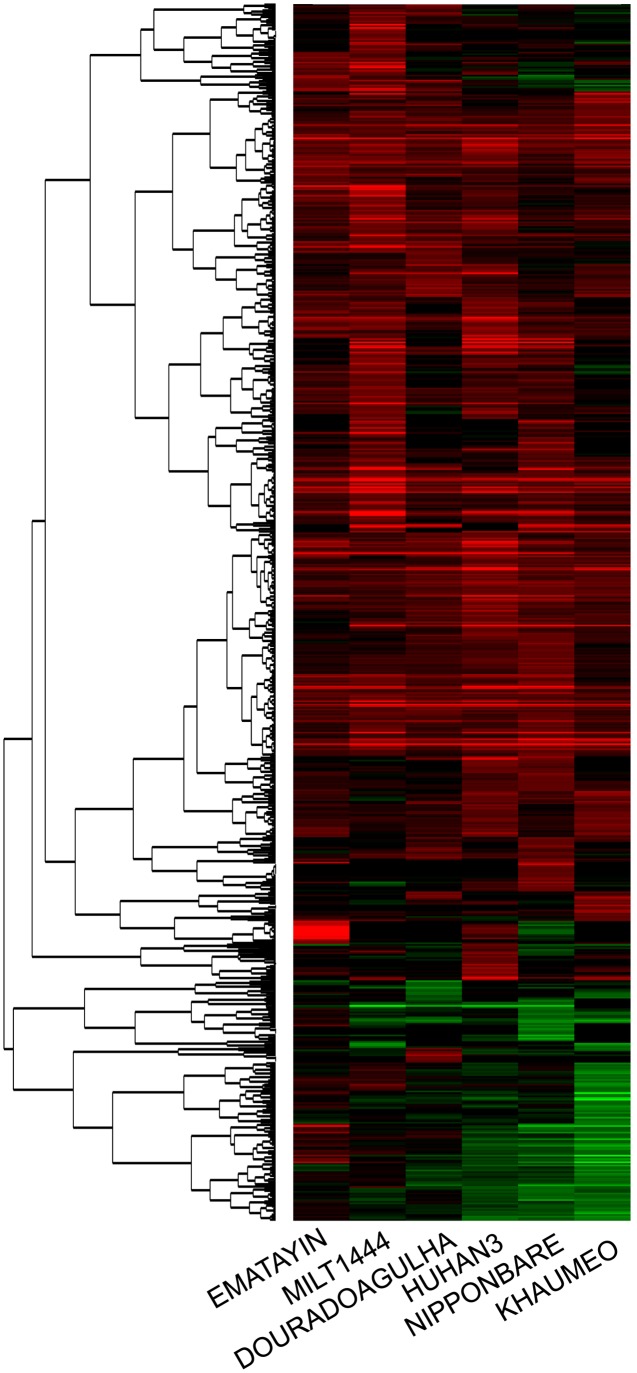
**Clustering results of differentially expressed genes in the microarray experiment**. Each row represented log2-fold change of the relative gene expression. Up-regulated expression genes were colored red, down-regulated expression genes were colored green, and black represented no significant expression.

To investigate the intrinsic differences in gene expression level between the drought tolerance and drought sensitive varieties, gene expression profiles at the tillering stage were evaluated with Affymetrix GeneChip. We obtained an average call rate 42.5% among the 57k microarray probe sets from six varieties, suggesting a good level of hybridization in our experiments. DEGs were identified by comparison between well watered and drought stress conditions for each rice variety. For DEG analysis, the criteria of differential expression was more than twofold changes and *p*-value < 0.05 based on three independent biological replicates. In total, 783 DEGs were identified to be drought-induced from young panicle in 2 cm length among six rice varieties (Supplementary Table S1). Importantly, panicle DEGs exhibited that the major genes were all expressed as up-regulated for each rice variety. There were 268 and 249 genes highly up-regulated (fold change ≥ 2) in MILT1444 and HUHAN3, which were more than those in the other four varieties (Supplementary Table S2). There were 140 and 52 genes significantly down-regulated (fold change ≤ 0.5) in drought sensitive KHAUMEO and NIPPONBARE, which were more than those in drought tolerant varieties (Supplementary Table S2). The detected DEGs in six rice varieties were subjected to cluster analysis. As shown in **Figure 3**, hierarchical clustering indicated that 76.8% of DEGs were up-regulated for all six rice varieties, and the percentage of down-regulated genes was higher in sensitive rice than tolerant varieties. We noted that the percentage of DEGs common to all six varieties is low, accounting for 3% (Supplementary Table S3). The result indicated that more genes were positively regulated by up-regulation expression as compared to down-regulated genes.

### Validation of DEGs by qRT-PCR

**FIGURE 4:**
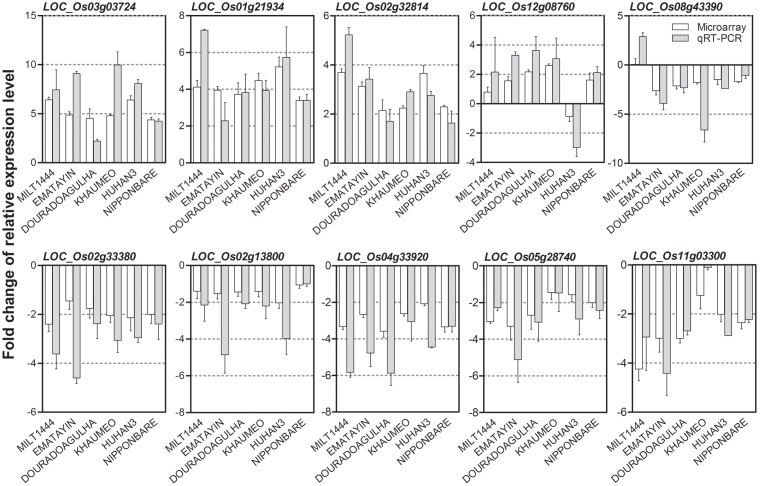
**Validation of reproducibility of drought-induced differentially expressed genes (DEGs) by qRT-PCR**. Ten randomly selected DEGs were performed qRT-PCR in six rice varieties. The bar graph shows relative fold change of each gene between drought stress and well watered conditions in different comparisons (labeled below the lower panel) obtained by qRT-PCR (white bar) and microarray (gray bar). The correlation coefficient between qRT-PCR and microarray data ranged from 0.91 to 0.99 in six rice varieties.

To validate the reproducibility of microarray gene expression results, quantitative real-time reverse transcription-PCR (qRT-PCR) was performed to assess the differential expression levels of 10 randomly selected DEGs in six rice varieties. The fold change of relative expression level between drought stress and well watered conditions were compared with gene microarray and qRT-PCR (**Figure 4**). Consistently, the qRT-PCR produced results were coincided very strongly with that from the microarrays. Genes *LOC_Os12g08760* and *LOC_Os08g43390* showed profoundly expression patterns with converse expression trends in one of varieties, but qRT-PCR results revealed a good agreement with gene microarray. The correlation coefficient between qRT-PCR and microarray data ranged from 0.91 to 0.99. These results indicated that the drought-induced DEGs from microarray are highly reliable.

### Functional Features of DEGs in Drought Tolerant and Drought Sensitive Varieties

The differences in phenotypic traits and drought-induced DEGs were observed for contrasting varieties as shown in above interpretation, which is consistent with their large genetic difference. To determine the similarities and differences in functional features of drought-induced DEGs for contrasting varieties, 783 DEGs were classified into drought-tolerant, drought-sensitive, and common-induced subsets, respectively. The DEGs in common-induced subset (46%) were significantly higher than that in drought-tolerant (29%) and drought-sensitive subsets (25%). The functional enrichment analysis by AgriGO indicated that the shared biological processes in three gene subsets were involved in response to stimulus, abiotic stimulus and stress. In addition, the distinct biological processes highly specialized for drought tolerant varieties were identified for GO terms, associated with regulation of biological quality, homeostatic process, cell growth, anatomical structure morphogenesis and development. The unique terms under the biological process category for drought sensitive varieties were identified as lipid metabolic process and secondary metabolic process. The biological process terms from three gene subsets revealed that both shared and distinct functional roles mediated stress-responsive genes for each group.

### Functional Mechanism of Drought-Induced DEGs in Young Panicle

**FIGURE 5:**
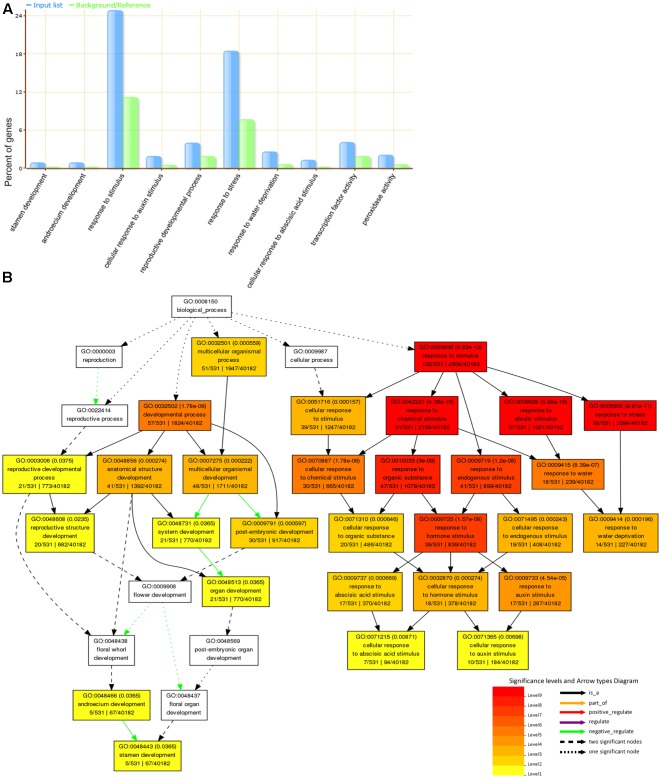
**Go enrichment analysis for drought-induced DEGs. (A)** Ten significant Gene Ontology (GO) terms were displayed, including reproductive developmental process, stamen development, response to stimulus, response to stress, water deprivation, abscisic acid stimulus, and so on. **(B)** The functional categories of biological processes of GO terms were shown as a diagram. Functional enrichment of drought-induced genes suggested two categories reproductive development and response to stimulus/stress were tightly related to protect panicle development against drought stress.

The incorporation of a mechanism to account for these drought-induced DEGs became necessary due to less enrichment category and less difference in panicle characters observed between tolerant and sensitive varieties. On the whole, functional enrichment revealed that 783 drought-induced DEGs were significantly associated with GO terms: stress response, reproductive developmental process, oxidation reduction (**Figure 5A**). The significant GO terms related to reproductive development and stress response were separately displayed (**Figure 5B**).

The genes related to floral development (such as GO: 0022414, 0048438, 0048437, and 0048443) were negatively regulated by drought stress (**Figure 5B**). Abnormal stamen development affected pollen fertility so that the floral developmental process was reduced, which decreased the rate or extent of development of flowerlets (Jin et al., 2013). The negative regulation by drought stress was corresponding to the result in the reduction of secondary branches per panicle, seed setting rate and grain weight (**Figure 2**). The latest results found that SAPK9 (LOC_Os12g39630), belonging to GO:0009791 and GO:0009414 terms, increased drought tolerance and grain yield by adjusting osmotic potential and stomatal closure (Dey et al., 2016). MADS-box transcription factors in plants play an important role in flowering and floral organ identity, as well as stress-related developmental processes. MADS26 (LOC_Os08g02070) was verified that it acts as an upstream regulator of stress-associated genes and a hub to modulate the response to various stresses in the rice plant (Khong et al., 2015). Our data also revealed OsMADS26 was located at GO:0009791 and GO:0048513 (**Figure 5**) and up-regulated by drought stress in MILT1444, HUHAN3, and NIPPONBARE. Additionally, the expression of several auxin-responsive genes in panicle were also significantly altered, such as OsIAA18 (LOC_Os05g44810), OsIAA26 (LOC_Os09g35870), OsSAUR20 (LOC_Os04g51890), OsSAUR31 (LOC_Os08g02520), OsSAUR33 (LOC_Os08g35110). SAUR proteins promote cell elongation (Spartz et al., 2014). OsIAA26 was also involved into negative regulation of stamen development, and OsGRX8 (LOC_Os02g30850) was induced by various stress conditions and plant hormone auxin (Sharma et al., 2013). Both of them were located at GO: 0048443 (**Figure 5**), and exhibited conserved function in floral organ development and stress response. These results suggested that auxin biosynthesis and transport in panicle were affected by drought stress. This might bring out the decrease of the length of panicle and secondary branches per panicle. Functional enrichment analysis suggested a significant association between functional categories of reproductive development and response to stimulus/stress. The interaction of reproductive and stress-responsive genes could protect panicle development against extreme drought environments.

### Genetic Associations with Drought Index Trait by the Gene-Based Association Analysis

In order to investigate genotypic differences in natural variation in drought tolerant and sensitive varieties, the gene-based association analysis was conducted using 65510 SNPs (MAF ≥ 0.05) from 783 drought-induced DEGs. The genotyping and the major agronomic traits including drought indexes in 270 rice accessions have been investigated in our previous study, and then were tested using a mixed linear model (MLM) in Tassel. Based on a threshold of -log(P) ≥ 4.0, 1445 SNP loci derived from 350 genes exhibited significant associations with drought index (Supplementary Table S4). We could found four associated genes, including LOC_Os01g50700, LOC_Os01g50910, LOC_Os02g13800, and LOC_Os02g46030, were consistent DEGs in six rice varieties. The genetic variation of four genes displayed distinct differentiation of *indica* and *japonica*. However, the fold change of gene expression did not show the distinct correspondence relationship with genetic variation between *indica* and *japonica*. The favorable SNP markers and candidate genes could narrow down the list of DEGs and provide useful resources for breeders.

## Discussion

Plants would be vulnerable to harm from water deficit at the reproductive stage. Researches on panicle have indicated the close relationship between grain yield and drought stress (Wang et al., 2011; Jin et al., 2013). This study provided new insight into the distinct processes of drought response in rice young panicle and the potential genetic variation for adaptation to drought at the genome-wide level.

Water deficit at the reproductive stage is crucial to panicle development, grain setting, filling, and final grain yield (Liu et al., 2010). The genetic variability of drought tolerance in varieties exhibited different panicle characters under drought stress (**Figures 1**, **2**). As the most important breeding goal, grain yield components is absolutely necessary to be selected and evaluation under stress environments. It’s reported that grain yield was positively correlated with panicle length/number, grain weight per panicle, seed setting rate, etc., nevertheless grain yield of rice is the integration of many variables throughout the growing period (Xu et al., 2015). The phenotypic evaluations of panicle between tolerant and sensitive varieties indicated reduction of parts of components must not result in decrease of grain yield. In MILT1444, panicle length and primary/secondary branches per panicle were decreased under drought stress, while seed setting rate was increased due to a longer delayed flowering time in response to drought treatment as compared to delay flowering of other five varieties. The seed setting rate under drought stress was higher than that under well watered condition, when the level of severity of drought stress in fields was mild (Kanjoo et al., 2012). Moreover, the most up-regulated genes among six varieties were positively involved in rice adaptive response to drought stress in MILT1444 (**Figure 3**). These findings revealed that modulated flowering time of rice in response to drought stress was directly correlated with positive regulation of up-regulated genes.

The expression level changes of drought-induced genes were also characterized for their panicle development, enabled us to correlate the phenotypic observations with the subjected biological processes (Su et al., 2013). Considering the less difference in panicle characters and enrichment category observed between tolerant and sensitive varieties, the incorporation of a mechanism to account for these drought-induced DEGs became necessary. The predominant DEGs were involved in distinct functional groups: response to stimulus and developmental process, including abiotic stimuli, water deprivation, abscisic acid stimulus, auxin stimulus, reproductive development, organ development, stamen development, and so on (**Figure 5**). The temporal-spatial gene expression profiling analysis from drought tolerant rice varieties revealed stress-responsive genes including many transcription factors were regulated in a tissue- or stage-specific manner (Wang et al., 2011; Harrop et al., 2016). MYB1 (LOC_Os08g33660.1) was thought to be induced by abiotic stress (Baldoni et al., 2015), its expression level was lesser in sensitive KHAUMEO than tolerance varieties under drought stress. Moreover, we found that several genes encoding proline-rich proteins were down-regulated in drought sensitive KHAUMEO and NIPPONBARE, indicating that proline-rich protein is critical for development and abiotic stress tolerance (Zhan et al., 2012). These results suggested the regulatory processes of drought-induced genes were tightly related to reproductive development and response to stimulus/stress, which protected panicle development against drought stress.

An important goal of this study was to dissect genes underlying the genotypic differences in adaption to drought environment. Natural variation in crops is an important way to adapt the development of environment (Juenger, 2013; Wei et al., 2016a). In natural populations, many studies were performed to measure genetic variation of crop traits and yield in response to drought (Liu et al., 2010; Yu et al., 2012; Lou et al., 2015). We have collected hundreds of rice accessions to be used for the water-saving and drought-resistance rice (WDR) breeding program, and obtained the basic agronomic traits and grain yields (Luo, 2010; Wu et al., 2015; Wei et al., 2016b). By narrowing down the list of DEGs based on the gene-based association analysis, 350 DEGs with significant association SNPs were also enriched in response to drought stress. Four associated genes exhibited consistently up-regulated in six rice varieties, but their genotypes were divided into two group corresponding *indica* and *japonica*. We supposed that these associated DEGs might be evolving under positive selection and positively involved in rice adaptive response to drought stress. Although these results displayed no significance between tolerant and sensitive varieties, these DEGs with significant SNPs gave valuable reference for rice breeding.

In summary, our study provided an elaborate comparison on panicle characters and gene expression profiles between tolerant and sensitive varieties in drought response. The majority of DEGs was up-regulated and had a positive impact on rice adaption to drought stress. High percentage of drought-induced DEGs was found to be significantly associated with SNPs, which provided a valuable direction for elucidation of genotypic differences in drought responses.

## Materials and Methods

### Plant Growth Conditions and Drought Stress Treatments

Seeds of six rice varieties, MILT1444, EMATAYIN, DOURADOAGULHA, KHAUMEO, HUHAN3, and NIPPONBARE, were collected from Shanghai Agrobiological Gene Center, China. Among of them, MILT1444, EMATAYIN, HUHAN3, and NIPPONBARE were *japonica* varieties with elite drought tolerance except for NIPPONBARE, while the others were *indica* varieties which the drought tolerance of DOURADOAGULHA is higher than of KHAUMEO (**Table 1**). Plants were grown in cement enclosed plots. Water was drained for drought stress in the plots at later tillering stage and allowed to dry. The control plants were grown in the same pool and watered well. After 2 weeks the soil water content decreased to 13.8%, panicle initiation and development was determined by manual measurement of rice panicle length. When young panicles were ∼2 cm in length corresponding to pollen mother cell formation stage, many genes were the highest expression in panicles with an expression peak, such as SG1 (Nakagawa et al., 2012), LABA1 (Hua et al., 2015), PMS1T (Fan et al., 2016). The developing young panicles (2 cm in length) were sampled and harvested into liquid nitrogen to store at -80°C for RNA isolation. The characters, including panicle length, seed setting rate, primary/secondary branches of per panicle and grain weight of per panicle, were obtained by manual measurement after harvest (**Figure 2**). Three independent experiments were carried out under drought stress and well watered conditions. The statistical *t*-test was used to evaluate significant difference of the morphological traits under drought and control conditions.

### RNA Extraction and Microarray Experiments

RNA isolation, purification and microarray hybridization were conducted by the CapitalBio Corporation (Beijing, China) according to the Affymetrix standard protocols. Gene expression microarray (4 × 44K format) was designed with 57381 probe sets, based on the latest genome annotation of *Nipponbare*^1^. Two microgram of the labeled sample RNA was used for microarray hybridization. Three independent experiments for microarray were conducted for each rice varieties under drought stress and well watered conditions, respectively.

### Real-Time PCR

Gene expression from microarray was validated by Quantitative Real-Time PCR (qRT-PCR). cDNA was synthesized from six rice varieties using a PrimeScript RT reagent kit with gDNA eraser (Takara, Dalian, China). qRT-PCR was performed using an AB7300 instrument (Applied Biosystems, Foster City, CA, USA) following the manufacturer’s instruction. Primers were designed using Primer3^2^ and synthesized by Sangon Biotech. These genes were randomly selected for DEGs set, and their primers were listed in Supplementary Table S5.

### Differentially Expressed Genes and Function Enrichment Analysis

The microarray data were compiled into gene expression data using the Affy package’s Robust Multichip Average (RMA) algorithm. Differential gene expression was assessed between replicate groups in drought stress and watered well using limma package on the Bioconductor (Ritchie et al., 2015). After being adjusted for multiple testing using the False Discovery Rate (FDR), DEGs were filtered by a FDR ≤ 0.05 and fold change ≥ 2. For function enrichment analysis, the identified DEGs was conducted GO analysis (Ashburner et al., 2000) using the online tool agriGO (version 1.2) (Du et al., 2010). The significant enrichment was determined by a Bonferroni adjustment to the adjusted *P* ≤ 0.01. Subsequently, hierarchical clustering was conducted from DEGs using Cluster 3.0, and clustering result was displayed using TreeView 3.0.

### Gene-Based Association Analysis

To examine whether genetic variants in these DEGs harbored association signals with the index of drought resistance, we performed a gene-based association analysis using our previously drought tolerance traits (Yu et al., 2012) and genome resequencing data (Wu et al., 2015). The low frequency alleles were filtered with MAF ≥ 0.05, and then 65510 SNPs with high quality were obtained for these drought-induced DEGs. The gene-based association analysis was conducted for the trait of index of drought tolerance using the general linear model (GLM) in Tassel 5.0 (Bradbury et al., 2007), from which significant SNPs with *P* ≤ 10^-4^ were selected for candidate gene analysis.

## Availability of Data and Material

The microarray data have been deposited in Gene Expression Omnibus (GEO) in NCBI under accession number GSE83378. The raw Illumina sequencing data from 270 rice accessions have been submitted to NCBI Sequence Read Archive (SRA) under the Bioproject number PRJNA171289 (Huang et al., 2012) and PRJNA260762 (Wu et al., 2015).

## Author Contributions

HW and SY designed the experiments and together with, CC and XM carried out gene microarray analysis, gene-based association analysis. CC, YZ, JH, and HM carried out rice morphological investigation, validation of gene expression level, functional analysis. HW was mainly involved in preparation of manuscript. All authors reviewed and approved the final manuscript.

## Conflict of Interest Statement

The authors declare that the research was conducted in the absence of any commercial or financial relationships that could be construed as a potential conflict of interest.

## Footnotes

1 http://www.affymetrix.com/products_services/arrays/specific/rice.affx

2 http://frodo.wi.mit.edu/
